# Biological Properties of a Novel Multifunctional Host Defense Peptide from the Skin Secretion of the Chaco Tree Frog, *Boana raniceps*

**DOI:** 10.3390/biom10050790

**Published:** 2020-05-20

**Authors:** Carlos José Correia Santana, Ana Carolina Martins Magalhães, César Augusto Prías-Márquez, Diego A. Falico, Agenor C. M. dos Santos Júnior, Beatriz D. Lima, Carlos André Ornelas Ricart, Denise Regina Bairros de Pilger, Rafaela Milan Bonotto, Carolina Borsoi Moraes, Lúcio H. Freitas-Júnior, Alice da Cunha Morales Álvares, Sonia Maria Freitas, Isabelle S. Luz, Osmindo Rodrigues Pires Jr., Wagner Fontes, Mariana S. Castro

**Affiliations:** 1 Laboratory of Toxinology, Department of Physiological Sciences, Institute of Biology, University of Brasília, Brasília 70.910-900, Brazil; carlosjcsantana@gmail.com (C.J.C.S.); bioana.11@gmail.com (A.C.M.M.); caprias@gmail.com (C.A.P.-M.); dialefalico@gmail.com (D.A.F.); osmindo@unb.br (O.R.P.J.); 2 Laboratory of Protein Chemistry and Biochemistry, Department of Cell Biology, Institute of Biology, University of Brasília, Brasília 70.910-900, Brazil; agenor.unb@gmail.com (A.C.M.d.S.J.); ricart@unb.br (C.A.O.R.); isabelle.sluz@gmail.com (I.S.L.); wagnerf@unb.br (W.F.); 3 Laboratory of Gene Biology, Department of Cell Biology, University of Brasília, Brasília 70.910-900, Brazil; beatrizdolabela@unb.br; 4 Department of Microbiology, Institute of Biomedical Sciences, University of São Paulo, São Paulo 05.508-900, Brazil; denise.pilger@gmail.com (D.R.B.d.P.); rafaela.bonotto@yahoo.com (R.M.B.); carolinaborsoi@gmail.com (C.B.M.); luciofreitasjunior@gmail.com (L.H.F.-J.); 5 Laboratory of Biophysics, Department of Cell Biology, Institute of Biology, University of Brasília, Brasília 70.910-900, Brazil; pharmalice@gmail.com (A.d.C.M.Á.); nina@unb.br (S.M.F.)

**Keywords:** amphibian, *Boana raniceps*, skin secretion, multifunctional defense peptide, antimicrobial peptide, structural analysis, neutrophils

## Abstract

In recent years, the number of new antimicrobial drugs launched on the market has decreased considerably even though there has been an increase in the number of resistant microbial strains. Thus, antimicrobial resistance has become a serious public health problem. Amphibian skin secretions are a rich source of host defense peptides, which generally are cationic and hydrophobic molecules, with a broad-spectrum of activity. In this study, one novel multifunctional defense peptide was isolated from the skin secretion of the Chaco tree frog, *Boana raniceps.* Figainin 2 (^1^FLGAILKIGHALAKTVLPMVTNAFKPKQ^28^) is cationic and hydrophobic, adopts an α-helical structure in 50% (*v/v*) trifluoroethanol (TFE), and is thermally stable. This peptide exhibited activity against Gram-negative and Gram-positive pathogenic bacteria arboviruses, *T. cruzi* epimastigotes; however, it did not show activity against yeasts. Figainin 2 also showed antiproliferative activity on cancer cells, is moderately active on human erythrocytes, and activates the oxidative burst in human neutrophils.

## 1. Introduction

The growing of antimicrobial resistance is a global public health problem. As the number of resistant microorganisms increases, the number of commercially available drugs to face them decreases dramatically: whereas, in the early 1980s, nineteen new drugs were launched, between 2010 and 2012, only one new drug was developed [1,2]. Recently, Laxminarayan and coauthors pointed out that 14 new antibacterial drugs have been approved in the past decade; however, there are gaps for combating Gram-negative microorganisms which are responsible for infections of medical significance, such as nosocomial infections [3]. The indiscriminate use of antibiotics in hospitals, agriculture, and self-medication has been contributing to an increasing prevalence of pathogenic multidrug-resistant organisms around the world [4]. On a global scale, it is estimated that approximately 214,000 neonatal deaths due to sepsis will occur annually caused by resistant microorganisms [5].

The emergence of ultra-resistant microorganisms is a serious health danger for the human population [6]. Colistin is one of the last-resort antibiotics for carbapenem-resistant bacteria; however, recently, a carbapenem-resistant and colistin-resistant *Escherichia coli* strain expressing NDM-9 and MCR-1 genes was identified in China [7]. In addition, these genes located on conjugative plasmids have been spreading to other bacteria as *Aeromonas* spp. [8] and *Klebsiella pneumoniae* [9].

Antimicrobial peptides (AMPs) or host defense peptides (HDPs) emerge as an alternative to face infections by multidrug-resistant microorganisms. These molecules are found in multicellular organisms and comprise their first line of defense. They are generally cationic and hydrophobic and showed variable size, between 10 to 50 amino acids long [10]. The activity of AMPs usually is not mediated by interactions between specific receptors but by direct interaction between the peptide and phospholipids of the plasma membrane [11].

Differences in plasma membranes between eukaryotic and prokaryotic cells contribute to the selectivity of AMPs. Mammalian cell membranes are composed by zwitterionic phospholipids, mainly sphingomyelin and phosphatidylcholine, whereas bacterial membranes are mostly composed of negatively charged phospholipids as phosphatidylglycerol and cardiolipin [12]. Direct interaction of AMPs with microorganism cell membranes promotes perturbation, disruption, pore formation, and death. In opposition, conventional antibiotics act in a limited number of molecular targets which are subject to mutation, consequently acquiring resistance [13].

In addition to antimicrobial activity, several amphibian peptides also showed activities against protozoans, cancer cells, and viruses as well as immunomodulatory effects [14,15]. The anticancer mechanisms involve cell membrane interaction, necrosis, and apoptosis [16]. On the other hand, the virucidal activity occurs by interaction with the viral envelope promoting membrane disruption, entry inhibition, and immunomodulation [14,17].

In the present study, we described the purification and characterization of a novel multifunctional host defense peptide isolated from the skin secretion of the frog *Boana raniceps* (Anura, Hylidae, Cophomantinae). This new peptide exhibited antibacterial, antiprotozoal, antiviral, anticancer, and immunomodulatory activities.

## 2. Material and Methods

### 2.1. Collection of the Specimens and Skin Secretion Harvesting

Adult specimens of *B. raniceps* were collected in Monte Alegre de Goiás in the state of Goiás, Brazil. The skin secretion was obtained by mild electrical stimulation and collected in a beaker by washing the animal skin surface with deionized water; it was then frozen and subsequently lyophilized and kept at −20 °C for subsequent use. The tree frogs were collected according to the Brazilian Environmental Agency (IBAMA—Instituto Brasileiro do Meio Ambiente e dos Recursos Naturais Renováveis) under the license number 51541-1. The skin-secretion-harvesting procedure was approved by the Animal Ethics Committee of the University of Brasília.

### 2.2. Peptide Purification

Aliquots of skin secretion (2.0 mg) were dissolved in 200 µL of solution A (trifluoroacetic acid (TFA) 0.1% (*v*/*v*) in Milli-Q water) and centrifuged for 10 min at 13,800 × *g*, and the supernatant was subjected to reversed-phase high-performance liquid chromatography (RP-HPLC) using a LC-20AT Prominence Liquid Chromatograph (Shimadzu, Kyoto, Japan) on a C_8_ column (Vydac 208TP54, 4.6 × 250 mm, Grace, CA, USA), previously equilibrated with solution A, and eluted according to the following gradient of solution B (TFA 0.1% (*v*/*v*) in acetonitrile): 0–25% of solution B in 5 min, 25–45% of solution B in 20 min, and 45–100% of solution B in 10 min at a flow rate of 1 mL/min and UV detection at 216 nm. Fractions were manually collected and dried in a vacuum concentrator. The fraction containing the peptide of interest was accumulated and injected into C_18_ RP-HPLC columns (Shim-pack VP-ODS 4.6 × 150 mm, Shimadzu, Kyoto, Japan or Vydac 218TP54, 4.6 × 250 mm, Grace, CA, USA), equilibrated with solution A, and eluted according to the following gradient of solution B: 0–55% of solution B in 5 min, 55–65% of solution B in 10 min, and 65–100% of solution B in 5 min at a flow rate of 1 mL/min and UV detection at 216 nm. All chromatographic runs were performed at ambient temperature (22 ± 2 °C).

### 2.3. Peptide Quantification

The peptide was quantified by spectrophotometry according to Aitken and Learmonth [18]. The peptide stock was aliquoted in a volume of 1 mL at 128 µM, was dried in a vacuum concentrator, and was stored at −20 °C until the moment of use. Each aliquot was resuspended in the same proportion using the respective buffer or medium for each experiment.

### 2.4. Structural Analysis

#### 2.4.1. Mass Spectrometry Analysis and Edman Degradation

Mass spectrometry analysis of the native peptide was performed in a Bruker Autoflex II TOF/TOF instrument (Bruker Daltonics, Bremen, Germany) in the reflected positive mode using α-cyano-4-hydroxycinnamic acid (HCCA) as matrix. The system was calibrated with peptide calibration standard II (Bruker Daltonics, Bremen, Germany). The peptide was resuspended in acetonitrile/water (1:1, *v*/*v*) containing TFA 0.1% (*v*/*v*), and 1 µL of peptide was applied to a stainless-steel plate with 1 µL of HCCA (10 mg/mL) and was analyzed in the *m*/*z* range of 550–4000. The native peptide was sequenced by automated Edman degradation on a Shimadzu PPSQ-33A (Shimadzu, Kyoto, Japan) according to the manufacturer’s protocols.

#### 2.4.2. Circular Dichroism Analysis

Circular Dichroism (CD) analysis was carried out using a Jasco J-815 spectropolarimeter (Jasco, Tokyo, Japan) equipped with a Peltier type temperature cuvette holder. Far-UV spectra of the peptide in Milli-Q water and in the presence of 10%, 30%, and 50% (*v*/*v*) trifluoroethanol (TFE) at 25 °C were recorded using 0.1 cm pathlength quartz cuvette. Spectra were analyzed at the wavelength range 190–260 nm. Four consecutive measurements were accumulated, and the mean spectra were corrected for the baseline contribution of the water and 10%, 30%, and 50% (*v*/*v*) TFE. Thermal denaturation assays were performed raising the temperature at a rate of 0.5 °C/min from 25 °C to 95 °C. The observed ellipticities were converted to molar ellipticity ([θ]) (deg.cm^2^.dmol^−1^) based on a mean molecular mass per residue of 115 Da. The α-helix secondary structure content was estimated considering the values of [θ]_208_ at different temperatures according to Greenfield and Fasman [19].

#### 2.4.3. Bioinformatics Analysis

The BLAST software (http://blast.ncbi.nlm.nih.gov/Blast.cgi) [20] and The Antimicrobial Peptide Database (APD, http://aps.unmc.edu/AP/database/query_input.php) were used to search for peptide sequence similarity; Expasy pI/Mw tool (http://web.expasy.org/compute_pi) [21] was used to determine the theoretical mass for the peptide; multiple sequence alignment was performed using Clustal Omega program (http://www.ebi.ac.uk/Tools/msa/clustalo/) [22]; helical wheel projection of the peptide was obtained using NetWheels software (www.lbqp.unb.br/NetWheels/) [23]; I-Tasser server was used to predict its secondary structure (https://zhanglab.ccmb.med.umich.edu/I-TASSER/) [24]; and the hydrophobic ratio and GRAVY (Grand Average of Hydropathy) were determined by APD3: Antimicrobial Peptide Calculator and Predictor (http://aps.unmc.edu/AP/prediction/prediction_main.php) [25].

### 2.5. Antimicrobial Assays

Initially, the fractions of two chromatographic runs were pooled and used in a qualitative screening assay against *Escherichia coli* (ATCC 25922) and *Staphylococcus aureus* (ATCC 25923). Each fraction was resuspended in 250 µL Milli-Q water and 50 µL of that solution was incubated with 50 µL of bacteria suspension. The bacterial suspensions were prepared as described below, and the assay was performed in duplicate. The samples were incubated at 37 °C for 22 h, and the absorbance was measured at 595 nm in a Multiskan FC microplate reader (Thermo Scientific, San Jose, CA, USA).

The bacteria used for the minimal inhibitory concentration (MIC) determination were strains of Gram-negative bacteria *Pseudomonas aeruginosa* (ATCC 27853), *Escherichia coli* (ATCC 25922), *Klebsiella pneumoniae* (ATCC 13883), and *Klebsiella pneumoniae* carbapanemase (KPC) multi-resistant clinical isolate (generously donated by Dr. Simoni Campos Dias from Catholic University of Brasília); Gram-positive bacteria *Enterococcus faecalis* (ATCC 29212), *Staphylococcus aureus* (ATCC 25923), *Staphylococcus epidermidis* (ATCC 12228), and *Enterobacter casseliflavus* (ATCC 700327). All bacteria strains were grown in Mueller–Hinton broth (MH) overnight at 37 °C under agitation. The bacterial suspensions were adjusted by addition of MH to OD_595_ = 1.0 and subsequently diluted 1:50 for Gram-negative and 1:100 for Gram-positive bacteria in fresh MH broth. Minimal inhibitory concentrations (MICs) were determined by microdilution method. Briefly, bacterial suspensions (aliquots of 50 µL containing 2 to 5 × 10^5^ colony-forming units depending on the bacterial strains used) were incubated in 50 µL of a serial dilution of the peptide (64 to 1 µM) dissolved in Milli-Q water. After incubation for 22 h at 37 °C, the optical density was measured at 595 nm using a Multiskan FC microplate reader (Thermo Scientific, San Jose, CA, USA). Each assay was performed in triplicate. For control, 50 µL of bacterial suspensions were incubated either with 50 µL of Milli-Q water or formaldehyde 0.8% (*v*/*v*). The MIC was defined as the lowest concentration of peptide or other antimicrobial agents at which no growth was detectable after incubation at 37 °C for 22 h.

For antifungal assay, strains of *Candida albicans* (ATCC 90028) and *Candida parapsilosis* (ATCC 22019) were grown in Brain Heart Infusion broth (BHI) for 24 h at 37 °C under agitation. The fungal suspensions were adjusted by addition of BHI to OD_595nm_ = 1.0 and then diluted in fresh BHI broth in the proportion of 1:100. Aliquots of 50 µL, containing approximately 2 × 10^5^ colony-forming units, were incubated in 50 µL of a serial dilution of the peptide (128 to 1 µM) dissolved in Milli-Q water. After incubation for 22 h at 37 °C, the optical density was measured in the same manner as previously described in the antibacterial assay.

### 2.6. Anti-Epimastigote Activity Against Trypanosoma cruzi

Epimastigote forms of *T. cruzi* (CL-Brener strain) were grown at 28 °C in Liver Infusion Tryptose (LIT) medium supplemented with 10% fetal bovine serum (FBS). Then, 100 µL of the parasite suspension at 5 × 10^6^ parasites/mL was incubated in a 96-well microplate with an equal volume of the peptide, serially diluted (64 to 0.25 μM) in LIT medium for 48 h to evaluate the inhibitory action on protozoan growth. After this time, each well received 40 μL of the CellTiter-Blue^®^ reagent solution (Promega, Madison, WI, USA) and was incubated for 4 h at 37 °C; then, the fluorescence (560_Ex_/590_Em_) was measured in a SpectraMax microplate reader (Molecular Devices, San Jose, CA, USA). The IC_50_ value was calculated by nonlinear regression using the program GraphPad Prism version 5.04.

### 2.7. Hemolytic Assay

Fresh human red blood cells (RBCs) were washed in 0.01 M Tris–HCl pH 7.4 containing 0.15 M NaCl (Tris-saline) three times at 500× *g* for 5 min and then resuspended 1% (*v*/*v*) in Tris-saline. Aliquots of 80 µL of serial 2-fold dilutions of the peptide dissolved in Tris-saline (at an initial concentration of 64 µM) were added to 80 µL of the 1% RBC suspension in microcentrifuge tubes. After 1 h at room temperature, the 96-well microplates with the samples were centrifuged at 500× *g* for 3 min. Aliquots of 80 µL of the supernatant were transferred to a new 96-well microplate, and absorbance was measured at 405 nm on a Multiskan FC microplate reader (Thermo Scientific, San Jose, CA, USA). Tris-saline and 1% (*v*/*v*) Triton X-100 served as controls. All peptide concentrations were tested in triplicate, and the data were expressed as mean ± SD. Percentage of hemolysis was calculated using the following formula:

% hemolysis = 100 (A_peptide_ − A_Tris-saline_)/(A_triton_ − A_Tris-saline_) (1)

The peptide concentrations that caused 50% lysis of RBCs (HC_50_) were calculated by nonlinear regression using the GraphPad Prism version 5.04 software. The RBC harvesting procedure was approved by the Human Ethics Committee of the University of Brasília.

### 2.8. Cytotoxicity Assay

B16F10 (ATCC CRL-6475) and MCF-7 (ATCC HTB-22) cells were cultured in Dulbecco’s modified Eagle’s medium (DMEM) with 10% FBS and antibiotics at 37 °C with 5% of CO_2_ as described previously [26]. The cell viability was determined by 3-4,5-dimethylthiazol-2,5 biphenyl tetrazolium bromide (MTT) (Molecular Probes, Thermo Fischer Scientific, Oregon, USA) assay. After 24 h of treatment with the peptide (64 to 0.5 µM), the cells were incubated with 15 µL of MTT 5 mg/mL in Phosphate-buffered saline (PBS) pH 7.4 and 135 µL of medium for 3 h; then, the medium was removed and 100 µL dimethyl sulfoxide (DMSO) was added. The absorbance was measured at 595 nm on a Multiskan FC microplate reader (Thermo Scientific, San Jose, CA, USA). The IC_50_ was calculated by nonlinear regression using the GraphPad Prism version 5.04 software.

### 2.9. Antiviral Assays

#### 2.9.1. Chikungunya Virus Assay

Huh 7 cells were seeded in a 384-well microplate with a density of 3000 cells/well. After 24 h, cells were infected with chikungunya virus (CHIKV) strain 181/25 with a multiplicity of infection (MOI) of 0.05. Compound samples were prepared as two-fold serial dilution, from 50 to 0.1 μM. Negative controls (0.5% DMSO and PBS infected cells), positive controls (50 μM 6-azauridine infected cells or noninfected cells with 0.5% DMSO) or compound samples were added to the respective plate wells. Then, plates were incubated for 48 h, fixed with paraformaldehyde (PFA) 3%, and conducted to immunofluorescence assay (IFA). Briefly, plates were incubated with a mouse hyperimmune serum (MIAF) anti-CHIKV and diluted in PBS with 5% FBS (1:1500) for 30 min. After two steps of washing with DPBS (Dulbecco’s phosphate-buffered saline) 1x, plates were incubated with the secondary antibody AlexaFluor488 conjugated goat anti-mouse IgG and 5 μg/mL of DAPI (4′,6-diamidino-2-phenylindole dihydrochloride). Then, plates were incubated for 30 min and washed again twice with DPBS 1x. Images and analyses were performed by IN Cell Analyzer 2200 GE. Images were taken from four different fields from each well at 20× magnification. The analysis consisted in identifying cell population from nuclei detection and infected cells from detected fluorescence signal in cytoplasm. The compound activity was measured based on infection ratio and total cell number values. Infection ratio (IR) was defined as the ratio between (i) the total number of infected cells in all images from the well and (ii) the total number of cells in all images from the same well. IR was normalized to negative (infected cells, DMSO-mock treated) and positive (noninfected cells) controls to determine the normalized activity. Cell survival was based on the ratio between average of cell number of 50 µM 6-azauridine per total cell number of wells tested. Selective index is the ratio between (i) CC_50_ (concentration which decreases 50% of cell survival) and (ii) EC_50_ (effective concentration of compounds which provided 50% viral inhibition).

#### 2.9.2. Dengue Serotype 4 Virus Assay (DENV4)

Stock compounds (1 mM) were serially diluted by a factor of 2 (i.e., in 2-fold serial dilutions) in H_2_O in 10 dilution points in a polypropylene 384-well microplate. For all tested samples, the highest concentration started at 50 µM, except for IFNα2A, which started at 1.3 nM. Then, 3 µL of compound solution were transferred from stock plates into black polystyrene 384-well assay microplates (Greiner BioOne, Kremsmünster, Austria), containing 7 µL of DPBS 1x, yielding a final concentration of 300 µM. Infections were performed with 1800 Huh7 cells/well co-plated with DENV4 at a MOI of 4.0 (resuspended in DMEM-F12 media), and 50 µL were dispensed in each well. Water and 1% DMSO were used as negative control, and infected cells treated with 5.2 nM interferon α 2A or noninfected cells were used as positive control. Then, plates were incubated for 72 h at 37 °C/5% CO_2_ under humidified atmosphere. Before the image acquisition at the imaging system IN Cell Analyzer 6000 (GE Healthcare, Illinois, USA), plates were fixed with 4% PFA, treated with 0.25% Triton X-100 for 5 min, and then stained with primary antibody against flaviviral E protein and then with secondary antibody goat anti-mouse conjugated with AlexaFluor488 and DAPI. The software Investigator (GE Healthcare, Illinois, USA) was optimized to gives us as readout multi-parameters: host cell number, infection ratio, and mean intensity of AlexaFluor488 signal per well. Infection ratio (IR) is the ratio between (i) the total number of infected cells in all images from the well and (ii) the total number of cells in all images from the same well. IR was normalized to negative and positive controls as described previously [27]. The cell ratio is an estimation of compound activity against the Huh7 host cell, and it is calculated to estimate compound selectivity towards DENV4.

#### 2.9.3. Yellow Fever Virus Assay

The Yellow Fever viral (YFV) strain 17D expressing the yellow fluorescent protein (YFP) was used to perform the assay as described for Dengue serotype 4 with few modifications. Briefly, infections were performed with 1800 Huh7 cells/well co-plated with YFV at a MOI of 2.5 (resuspended in DMEM-F12 media), and 50 µL was dispensed with the aid of a 16-multichannel pipette in each well. Then, plates were incubated for 72 h at 37 °C/5% CO_2_ under humidified atmosphere. Before the image acquisition, plates were fixed with 4% PFA and then stained with DAPI. The software Investigator (GE Healthcare, Illinois, USA) was optimized to gives us as readout multi-parameters host cell number, infection ratio, and mean intensity of YFP signal per well.

### 2.10. Neutrophil Phagocytosis and Oxidative Burst

Human neutrophils were isolated from the heparinized venous blood of four healthy donors using Percoll density gradients. After centrifugation, the cells were washed twice and residual RBCs were removed by hypotonic lysis. Only samples presenting >97% viable neutrophils (as determined by trypan blue exclusion) were used for the next steps. Neutrophils were divided in three aliquots of 6600 cell/µL and suspended in Hanks’ balanced salt solution (HBSS) with 185.4 mg/L calcium, 200 mg/L magnesium, and autologous plasma. Each aliquot was incubated for 30 min at 37 °C with the respective activator: 100 nM N-Formyl-L-methionyl-L-leucyl-L-phenylalanine (fMLP) (Sigma F3506), 8 µM Figainin 2, or HBSS (as negative control). To compare neutrophil oxidative burst capacity between conditions, nitroblue tetrazolium (NBT) conversion into formazan was assessed by optical microscopy. Neutrophils from the three activation systems were incubated with *Saccharomyces cerevisiae* for 40 min at 37 °C in a ratio of 5 yeast cells to 1 neutrophil. Right after incubation, cells were fixed on glass slides, stained with panoptical stain, and analyzed by optical microscopy.

## 3. Results and Discussion

### 3.1. Purification Procedure and Mass Spectrometry Analysis

**Figure 1:**
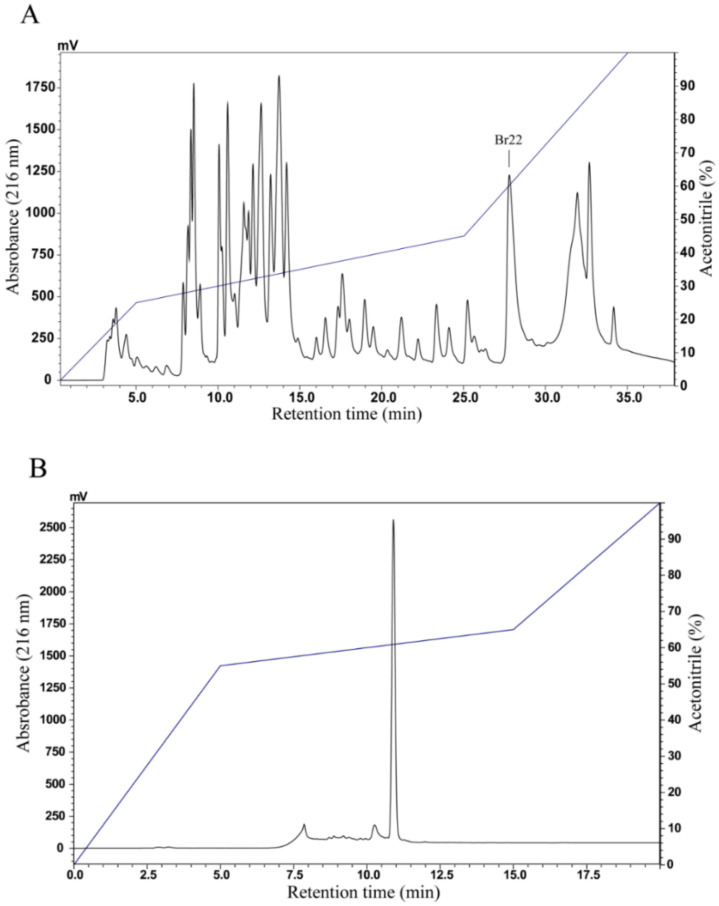
(**A**) Chromatographic profile of *B. raniceps* crude skin secretion fractionated by RP-HPLC performed on a Shimadzu LC system using a Vydac C_8_ column. (**B**) Rechromatography of the marked fraction (with antibacterial activity) by RP-HPLC using a Shim-pack VP-ODS C_18_ column.

**Figure 2:**
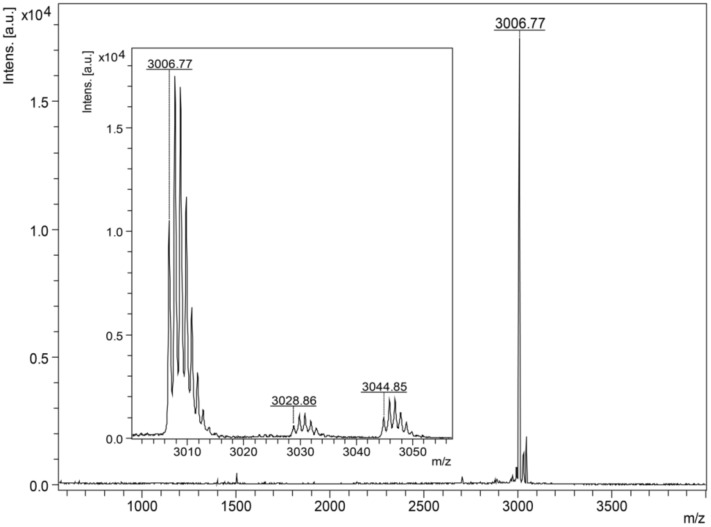
MALDI-TOF mass spectrum of Figainin 2. The insert showed the presence of sodium and potassium adducts +22 Da and +38 Da, respectively.

The skin secretion of *B. raniceps* was fractionated by RP-HPLC using a C_8_ column and resulted in a typical chromatographic profile with the elution of 25 fractions. An antibacterial screening assay with *E. coli* ATCC 25,922 and *S. aureus* ATCC 25,923 allowed the identification of an active fraction, labeled as Br22 in the chromatogram (Figure 1A). Rechromatography of this antibacterial fraction was performed using a C_18_ column (Figure 1B) and analyzed by matrix-assisted laser desorption/ionization-time of flight mass spectrometry (MALDI-TOF MS), showing that the molecular mass of the major component as 3006.77 Da [M + H]^+^ (Figure 2).

### 3.2. Sequence Analysis

**Figure 3:**
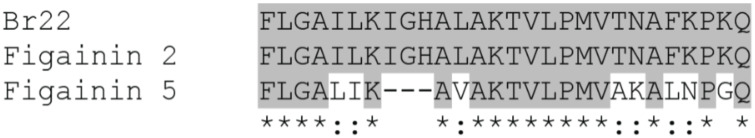
Sequence alignment of Br22 to other putative host defense peptides (HDPs) from *B. raniceps*: The highlighted letters and asterisk indicate identical amino acids.

The sequence of Br22 obtained by Edman degradation was ^1^FLGAILKIGHALAKTVLPMVTNAFKPKQ^28^, and similarity searches using BLASTp and APD showed that Br22 is identical to the mature peptide deduced from Figainin 2 precursor and has high similarity to the mature peptide deduced from Figainin 5 precursor, both identified in a cDNA library from *B. raniceps* skin but not yet isolated in the peptidic form [28] (Figure 3).

Figainin 2 is cationic (net charge +4) and has a hydrophobic ratio of 53% and a GRAVY score of 0.575 (Table 1). The GRAVY score was obtained by the sum of the hydropathy values divided by the number of amino acid residues [29]; positive indicates peptides with hydrophobic character, and negative indicates hydrophilic [30]. This peptide is rich in leucine, alanine, glycine, and lysine, which are abundant in host defense peptides from amphibian skin secretions [31].

### 3.3. Secondary Structure Prediction and Circular Dichroism Analysis

**Figure 4:**
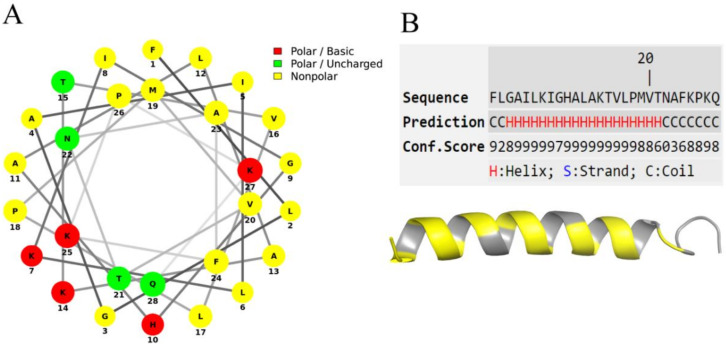
(**A**) Schiffer–Edmunson prediction of Figainin 2 showing the hydrophilic face and the nonpolar and hydrophobic face (in yellow). (**B**) Secondary structure model using I-Tasser server: Hydrophobic amino acids are marked in yellow.

The Schiffer–Edmunson prediction indicates that Figainin 2 adopts an amphipathic structure, with a hydrophobic side in opposition to the hydrophilic (Figure 4A), a typical feature of cationic host defense peptides [32]. The peptide projection at I-Tasser server results in 3D structure with a prevalent α-helix component and N- and C-terminal portions in coil form (Figure 4B).

**Figure 5:**
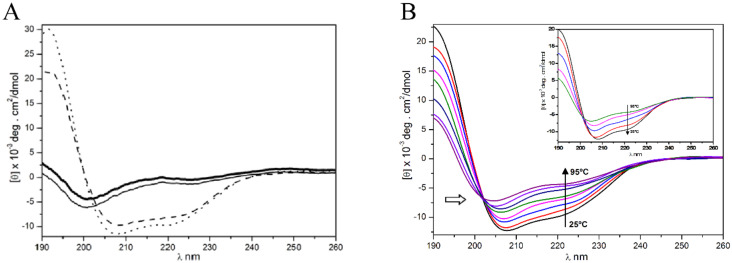
(**A**) Far-UV Circular Dichroism (CD) spectra of Figainin 2 at 25 °C. Solid intense black line shows the CD spectrum in water. Slim solid, dashed, and dotted lines represent the spectra in 10%, 30%, and 50% (*v*/*v*) trifluoroethanol (TFE), respectively. The red shift of molar ellipticity from 200 nm (solid line) to 208 and 222 nm (dotted and dashed lines) are observed as a function of TFE addition (10–50%). (**B**) Effect of temperature from 25 °C to 95 °C on secondary structure of Figainin 2 in TFE 50% (*v*/*v*): The decreased dichroic bands at 222 and 208 nm are shown as a function of temperature, indicating the changes of the α-helix structure contents (Table 3). The isodichroic point of about 203 nm is indicated by the arrow. Insert: Dichroic spectra of the renaturation process of Figainin 2 in TFE 50% (*v*/*v*).

The Far-UV CD spectra of Figainin 2 in Milli-Q water and 10% (*v*/*v*) TFE show random coil structure predominance, as indicated by the negative dichroic bands at 200 nm (Figure 5A). The α-helix spectra pattern was observed with addition of 30% and 50% (*v*/*v*) TFE, as indicated by the red shift and the intense negative dichroic band at 208 and 222 nm. 

This result suggests the tendency of Figainin 2 to adopt an α-helix folding pattern in hydrophobic/aqueous environments (Table 2). The Far-UV CD spectra recorded during the unfolding (from 25 °C to 95 °C) (Figure 5B) and refolding (from 95 °C to 25 °C) (Figure 5B, *insert*) of Figainin 2 in TFE 50% (*v*/*v*) show the decrease and increase, respectively, of dichroic bands at 208 and 222 nm, compatible with partial thermal stability of the peptide. The content of the α-helix structure decreased from 69.80% (25 °C) to 40.27% (95 °C) (Table 3). The renaturation of Figainin 2 after heating at 95 °C and decreasing temperatures of 85, 65, 45, 35, and 25 °C (Figure 5B, insert) was indicated by the spectra profiles returning from 95 °C to the initial state at 25 °C. Little change in secondary structure was detected, suggesting secondary structure rearrangement of the peptide depending on the temperature.

Additionally, the isodichroic point for Figainin 2 was observed near 203 nm (Figure 5B), suggesting equilibrium transition of the peptide between two conformational folding and unfolding states [33,34,35].

### 3.4. Antimicrobial Activity

The antimicrobial properties of Figainin 2 were evaluated against pathogenic Gram-negative and Gram-positive bacteria and yeasts (Table 4). Figainin 2 was effective against Gram-negative bacteria with MIC = 16 µM for *K. pneumoniae* KPC (multi-resistant clinical isolate strain), 8 µM for *E. coli* and *K. pneumoniae*, and 32 µM for *P. aeruginosa*. All Gram-positive bacteria tested were sensitive to Figainin 2: *S. epidermidis* and *E. casseliflavus* with MIC = 4 µM and *S. aureus* and *E. faecalis* with MIC = 8 µM. On the other hand, this peptide did not show activity against the yeasts *C. albicans* and *C. parapsilosis*.

Figainin 2 was more active against Gram-positive bacteria, and this property is similar to the ones exhibited by Hylin-a1 from *B. albopunctata* [36] and by HS-1 from *B. semilineata* [37], both peptides with hydrophobic ratio higher than 50%. Oppositely, the AMP Raniseptin 1 from *B. raniceps* which exhibits a less hydrophobic ratio of 44% is more active against Gram-negative bacteria [38].

The mechanism of action of cationic antimicrobial peptides involves the disruption of cell membrane by electrostatic interaction of cationic peptides with negatively charged phospholipids and acts in intracellular targets as proteins, DNA, and RNA synthesis [39]. Moreover, the nonspecific membranolytic mechanism of AMPs are similar against susceptible and resistant microorganisms and the broad-spectrum activity of these molecules represents an important source for the development of new antimicrobial drugs [39]. In a recent review, Koo and Seo highlighted that 27 peptides from different origins are under clinical studies for application on various targets such as bacteria and fungi infections [40]; however, to be pharmaceutically relevant, some properties of Figainin 2 should be improved including the selectivity against microorganisms and the cytolytic effects on RBCs. One alternative is to design a series of analogs using Figainin 2 as a template in order to improve the efficacy on different pathogens [15].

Although the mechanisms of action are similar between Gram-positive and Gram-negative bacteria, the differences between cell wall composition is an important factor and may influence the lytic activity of the peptide. Neelay et al. showed that the cationic peptides Cecropin A and Mellitin interact with peptidoglycan in a manner similar to vancomycin [41]. Peptidoglycan is one of the major components of the cell wall of Gram-positive bacteria, and our results show that the peptide Figainin 2 was more effective against the bacteria *S. epidermidis* and *E. casseliflavus*; therefore, it may act similarly to those peptides.

### 3.5. Antitrypanosomal Activity

The activity of Figainin 2 against epimastigote forms of *T. cruzi* was evaluated after 48 h at different concentrations and IC_50_ = 6.32 μM was obtained by nonlinear regression (Table 4). Figainin 2 showed a significant effect against these parasites, and in this concentration, the peptide shows no toxicity against eukaryotic cells. Several peptides isolated from amphibian skin secretions have been reported as antiparasitic agents [15]; however, there are few studies that address the possible mechanisms of action of AMPs in *Trypanosoma* species. Dermaseptins 1 and 4 and Phylloseptins 7 and 8 (isolated from *Pithecopus nordestinus*) are able to permeabilize the plasma membrane of the parasites *T. cruzi* and *Leishmania* [42]. Krugliak et al. showed that Dermaseptin S4 analogs act in erythrocytes infected with *Plasmodium falciparum* parasites, whereas uninfected cells were preserved [43]. The mechanism of action of antiprotozoal peptides appears to be mostly via pore formation and rupture of the plasma membrane; however, intracellular events such as mitochondrial membrane depolarization or DNA fragmentation were observed in *L. infantum* promastigotes after exposure to Temporin-SHa analogs [44].

### 3.6. Hemolytic Activity

**Figure 6:**
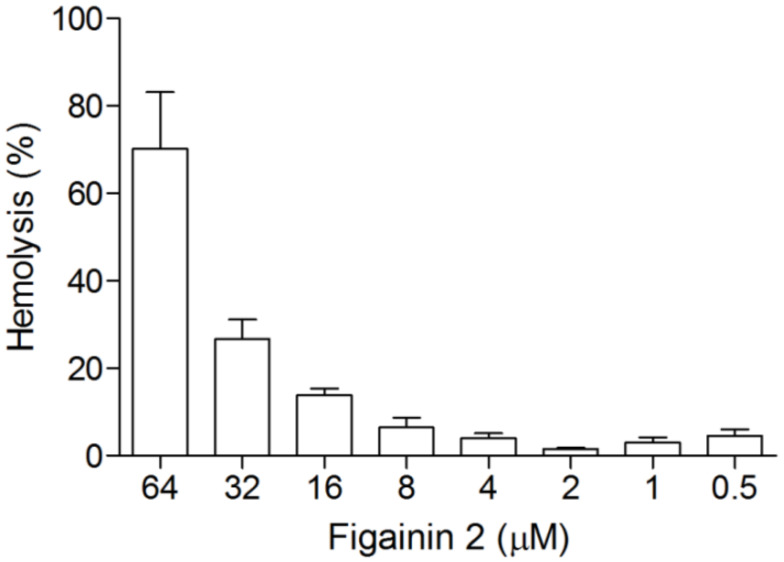
Hemolytic effects of Figainin 2 in human erythrocytes: Triton X-100 1% (*v*/*v*) was used as positive control (100% hemolysis), and Tris-saline was used as negative control (0% hemolysis). Data points show mean ± SD.

Hemolytic activity of Figainin 2 was evaluated using human RBCs (Figure 6). Figainin 2 displays an HC_50_ = 48.9 µM and promotes hemolysis at a moderate rate with 23% of hemolysis at 32 µM and 9% at 16 µM. With exception of *P. aeruginosa* (MIC = 32 µM), cytolytic activity in the range of effective concentrations that inhibits other tested bacteria does not induce a significant hemolysis.

The toxicity of AMPs in eukaryotic cells is a critical obstacle for the clinical application. Similarly, to microorganisms, the hemolytic activity of cationic peptides is related with multiple physicochemical parameters as charge, helicity, hydrophobicity, amphipathicity, and others [45]. The cationic peptide Mellitin, the predominat component of bee venom, displays potent antimicrobial and cytolytic activities with HC_50_ and MIC values below 5 µM [46]. On the other hand, the peptide Cecropin B, originally isolated from the moth *Hyalophora cecropia*, exhibits low toxicity against RBCs but is active on Gram-negative bacteria [47].

### 3.7. Antiproliferative Activity

**Figure 7:**
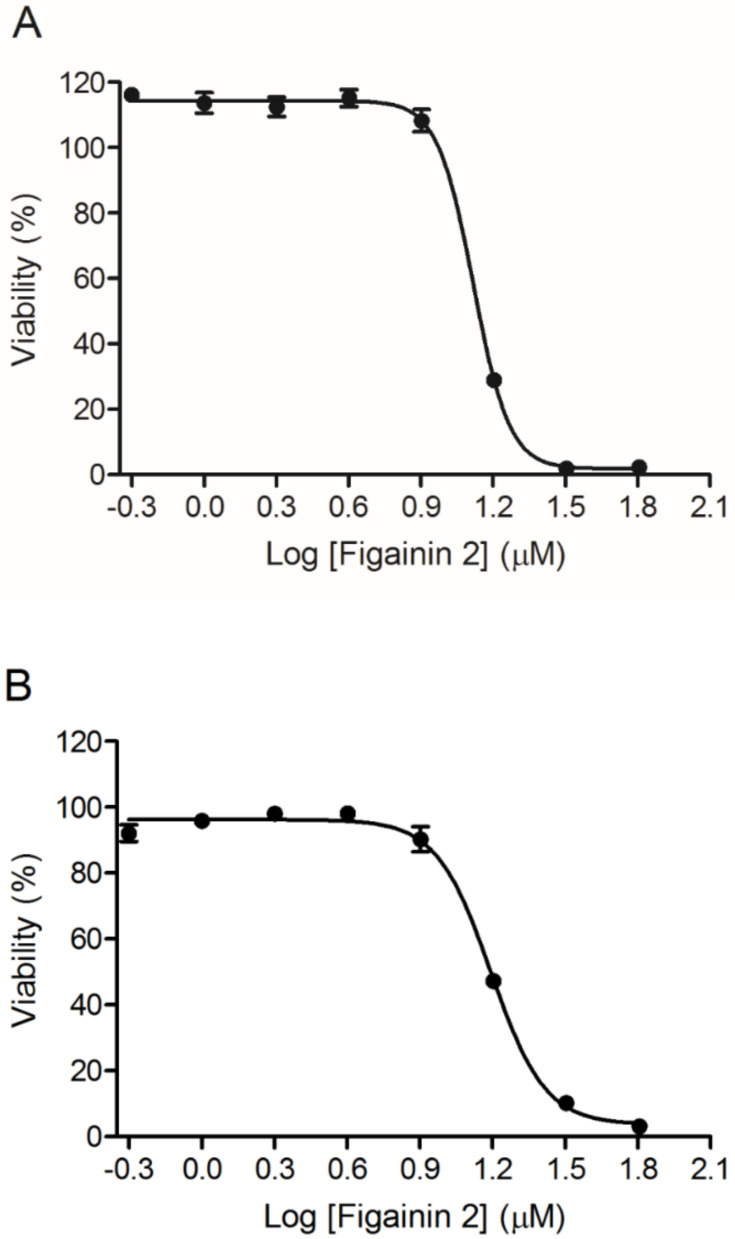
Cytotoxic activity of Figainin 2 against cancer cell lines (**A**) B16F10 (IC_50_ = 12.8 µM) and (**B**) MCF-7 (IC_50_ = 15.3 µM): Data points show mean ± SD.

The antiproliferative activity in MCF-7 and B16F10 cancer cells was evaluated in a range of concentrations from 64 to 0.5 µM after 24 h, and cell viability was measured by MTT assay (Figure 7). It was observed that Figainin 2 shows IC_50_ = 12.8 µM for B16F10 (murine melanoma cells) and IC_50_ = 15.3 µM for MCF-7 (human breast cancer cells) in a dose-dependent manner.

Recently, the use of host defense peptides as anticancer agents has increased considerably as an alternative in the development of new drugs. These peptides can act directly in the plasma membrane inducing pore formation, as Citropin 1.1 and Pexiganan MSI-7, or intracellular targets, as Brevinin-2R which induces caspase activation and reactive oxygen species generation [48] and Pentadactylin that promotes alteration of mitochondrial membrane potential, DNA fragmentation, and cell cycle arrest at the S phase and in B16F10 cells, indicating apoptosis [26].

### 3.8. Antiviral Properties

**Figure 8:**
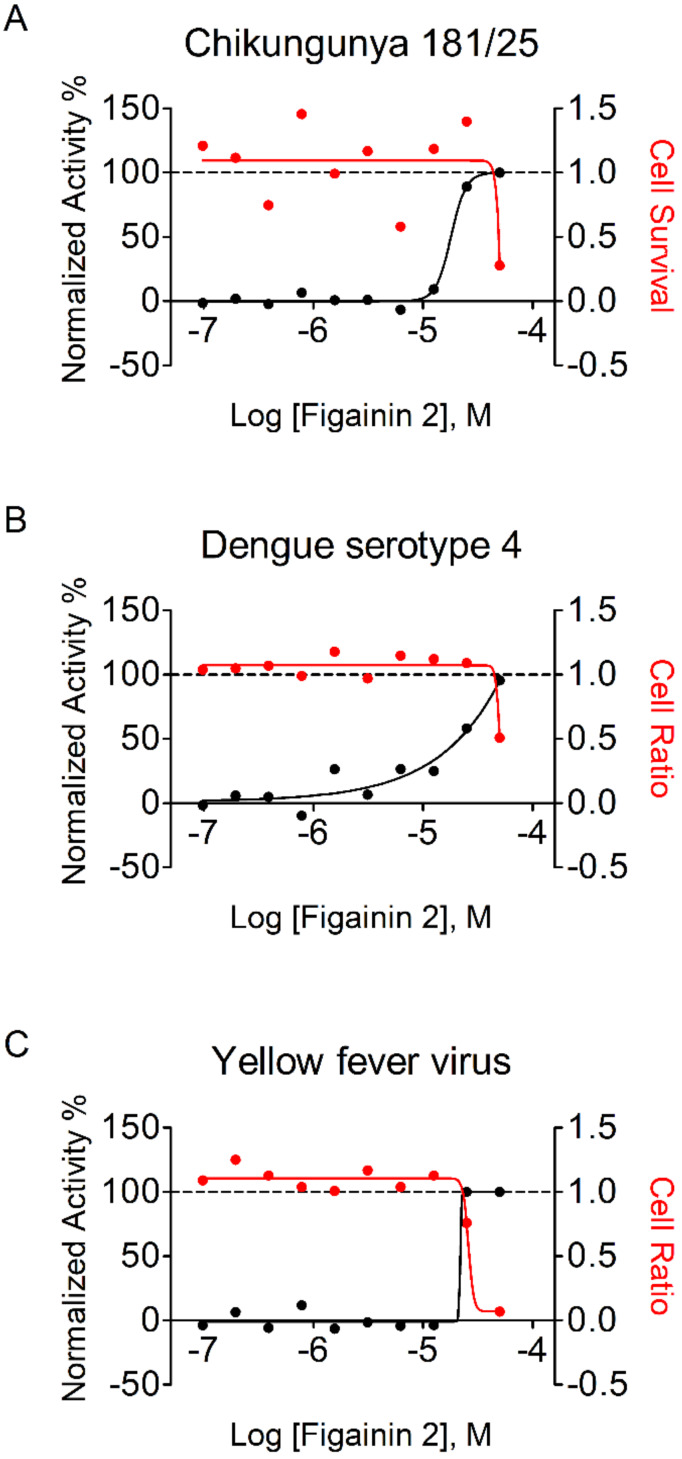
Dose-response curves of Figainin 2: The X-axis indicates the log of Figainin 2 concentration (molar); the right Y-axis shows the normalized antiviral activity in percentage (in black), which represents the inhibition of infection in relation to positive control; and the right Y-axis shows the cell ratio or cell survival (in red). (**A**) chikungunya virus (CHIKV) 181/25, EC_50_ = 17.9 μM, CC_50_ = 49.0 μM, and SI = 2.88; (**B**) Dengue serotype 4 virus, EC_50_ = 20.8 μM, CC_50_ = 50.1 μM, and SI = 2.41; and (**C**) Yellow Fever Virus, EC_50_ = 21.8 μM, CC_50_ = 26.4 μM, and SI = 1.21.

To determine the antiviral activity of Figainin 2, the peptide was tested against Huh7 cells infected with CHIKV 181/25, DENV4, or YFV. Our data showed that the peptide inhibited all viral infections in a dose-dependent manner (Figure 8), with a slightly higher potency for CHIKV (EC_50_ = 17 µM) in comparison with other tested viruses (EC_50_s of 20.8 and 21.8 µM for DENV4 and YFV, respectively). Furthermore, the peptide was not toxic for noninfected Huh7 cells at concentrations as high as 25 µM.

According to Shartouny and Jacob [17], the antiviral property of peptides can be either through inhibition of host cell entry mechanisms or through viral membrane disruption. The antiviral property of many frog-derived peptides has been reviewed by Conlon and colleagues [13]. As an example, the study done by VanCompernolle et al., 2005 [49], showed that three frog-derived peptides were able to reduce the HIV-1 infection without being toxic to the T cells. They also showed that antiviral activity was through the disruption of HIV membrane integrity upon peptide interaction [49]. Holthausen and colleagues characterized another frog-derived peptide that was specific for H1 influenza virus. The peptide neutralized the viruses by targeting the hemagglutinin proteins on the surface of the enveloped virions [50]. Although Figainin 2 did not exhibit significant antiviral activity against these viruses, our results lead us to believe that frog-derived peptides may be a viable source for antiviral therapies.

### 3.9. Modulatory Activity on the Innate Immune System

**Figure 9:**
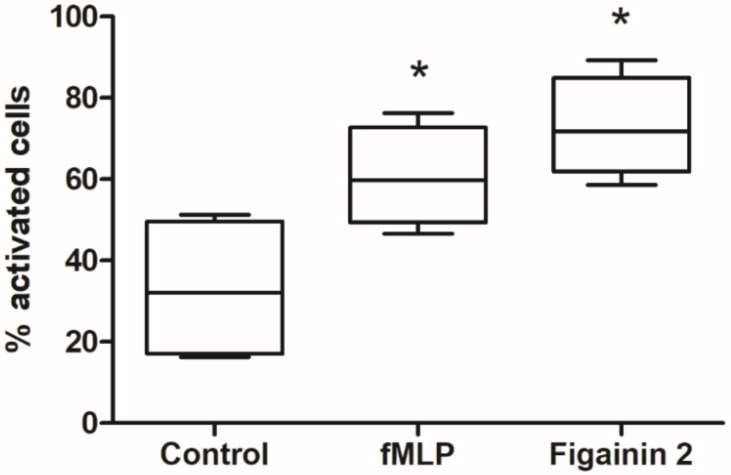
Reactive oxygen species (ROS) generation in human neutrophils in response to control (HBSS), fMLP (0.1 µM), and Figainin 2 (8 µM): Differences between the samples were determined by one-way ANOVA with Tukey’s posttest. * *p* < 0.05 compared to control.

Figainin 2 at the concentration of 8 μM induced the production of reactive oxygen species (ROS) by human neutrophils, as evidenced by the NBT test. Our data show that Figainin 2 in a subtoxic dose against mammalian cells had a similar effect to the bacterial peptide fMLP that was used as a positive control, significantly increasing the ROS levels in neutrophils (Figure 9). On the other hand, the peptide did not cause any significant change in the phagocytic activity of the cells (as opposed to the effect caused by fMLP), suggesting a specific immunomodulatory activity that triggers specific intracellular pathways, probably independent of the formyl peptide receptor family. In addition to activity against microorganisms, peptides have been reported as important effectors in the modulation of immune response in mammals.

The LL-37 human cathelecidin, in addition to having potent broad-spectrum antimicrobial activity, also modulates the activity of various cell types including chemotaxis and degranulation on mast cells and inhibition of pro-inflammatory cytokines and stimulates the production of ROS in neutrophils [51,52], whereas human beta-defensin (hBD2) induces mast cell degranulation and the production of prostaglandin D_2_ [53].

In amphibian skin secretions, several peptides with immunomodulatory properties have been isolated [48]. Temporin A isolated from *Rana temporaria* shows a potent chemoattractant effect in monocytes, neutrophils, and macrophages both in vitro and in vivo, and its action seems to occur via G protein-coupled receptor FPRL1 [54]. Dermaseptin S9 initially isolated from the tree frog *Phyllomedusa sauvagei* exhibits chemotatic activity for neutrophils, T lymphocytes and monocytes and their activity also appear to occur via FPRL1 [55]. Recently, two new peptides named AC12 and DK16 isolated from *B. raniceps* displayed immunomodulatory effects, significantly reducing the production of NO and of the cytokines TNF-α, IL-12, and IL-10 in lipopolysaccharide (LPS) stimulated RAW 264.7 macrophages, and in vivo, the peptide AC12 showed an anti-inflammatory effect in carrageenan-induced paw edema assay, reducing cell migration in the mouse paw tissue [56].

## 4. Conclusions

In this study, we described the isolation and characterization of a novel multifunctional host defense peptide from *B. raniceps* skin secretion. This novel peptide is identical to the mature peptide deduced from Figainin 2 precursor previously identified in a cDNA library obtained from *B. raniceps* skin. Figainin 2 is cationic and rich in hydrophobic amino acids and adopts an amphipathic α-helical structure in the presence of TFE. Our data demonstrate that Figainin 2 is a broad-spectrum host defense peptide active on Gram-negative and Gram-positive pathogenic bacteria and on emerging arboviruses, displays activity against *T. cruzi* epimastigotes and cancer cells, and exhibits immunomodulatory effects in human neutrophils.

## Figures and Tables

**Table 1:** Physicochemical properties of Figainin 2: The calculated molecular mass (calc) and the observed molecular mass (obs) are expressed for the deprotonated form.

Peptide	Mass Calc. (Da)	Mass Obs. (Da)	Net Charge	Hydrophobic Ratio (%)	GRAVY
Figainin 2	3005.76	3005.77	+4	53	0.575

**Table 2:** Content of α-helix structures of Figainin 2 in water and at different concentrations of TFE.

	Water	TFE 10%	TFE 30%	TFE 50%
Figainin 2	9.10	20.10	62.08	69.80

**Table 3:** Content of α-helix structures of Figainin 2 in TFE 50% (*v*/*v*) at different temperatures.

	25 °C	35 °C	45 °C	55 °C	65 °C	75 °C	85 °C	95 °C
Figainin 2	69.80	64.50	59.70	56.10	54.20	48.03	43.80	40.27

**Table 4:** Antimicrobial and anti-*T cruzi* activities of Figainin 2.

Microorganisms	Figainin 2 (µM)
**Gram-negative bacteria (MIC)**	
*E. coli* (ATCC 25922)	8
*P. aeruginosa* (ATCC 27853)	32
*K. pneumoniae* (ATCC 13883)	8
*K. pneumoniae* carbapanemase (KPC) MR	16
**Gram-positive bacteria (MIC)**	
*S. aureus* (ATCC 25923)	8
*E. faecalis* (ATCC 29212)	8
*S. epidermidis* (ATCC 12228)	4
*E. casseliflavus* (ATCC 700327)	4
**Yeast (MIC)**	
*C. albicans* (ATCC 90028)	NA
*C. parapsilosis* (ATCC 22019)	NA
***Trypanosoma* epimastigotes (IC_50_)** *T. cruzi*	6.32

MIC: minimal inhibitory concentration; MR: multi-resistant strain; NA: no activity at 128 µM and IC_50_: half maximal inhibitory concentration.
